# Reduced bone density accrual among peripubertal boys with HIV in Zimbabwe

**DOI:** 10.1097/QAD.0000000000004134

**Published:** 2025-02-27

**Authors:** Lisha Jeena, Rashida A. Ferrand, Victoria Simms, Cynthia Kahari, Tsitsi Bandason, Ruramayi Rukuni, Andrea M. Rehman, Sarah Rowland-Jones, Anthony Y.Y. Hsieh, Celia L. Gregson

**Affiliations:** a Oxford Centre for Immuno-Oncology, Nuffield Department of Medicine, University of Oxford, Oxford; b Clinical Research Department, Faculty of Infectious and Tropical Diseases, London School of Hygiene and Tropical Medicine, London, UK; c The Health Research Unit Zimbabwe, Biomedical Research and Training Institute, Harare, Zimbabwe; d MRC International Statistics and Epidemiology Group, Department of Infectious Disease Epidemiology, Faculty of Epidemiology and Population Health, London School of Hygiene and Tropical Medicine, London, UK; e Department of Oncology, Medical Physics and Imaging Sciences, Faculty of Medicine and Health Sciences, University of Zimbabwe, Harare, Zimbabwe; f Musculoskeletal Research Unit, Translational Health Sciences, Bristol Medical School, University of Bristol, Bristol, UK.

**Keywords:** bone density/content, dual X-ray absorptiometry, HIV, peripubertal children/adolescents, tenofovir disoproxil fumarate

## Abstract

**Objective:**

To investigate bone density accrual over 1 year among peripubertal children with HIV (CWH) compared to children without infection (CWOH); and risk factors associated with bone density accrual among CWH.

**Design:**

A prospective cohort study in urban Zimbabwe.

**Methods:**

CWH on antiretroviral therapy aged 8–16 years, and CWOH, frequency-matched by age were recruited in Zimbabwe. *Z*-scores for height-adjusted total-body less-head bone mineral content for lean mass (TBLH-BMC^LBM^) and size-adjusted lumbar spine bone mineral apparent density (LS-BMAD) were calculated from dual X-ray absorptiometry (DXA) scan measurements. Linear regression compared bone density accrual by HIV status.

**Results:**

Of 609 participants, 492 (80.7%) completed a follow-up visit (50.2% boys, 49.6% CWH). Mean baseline age was 12.5 years. More girl CWH than CWOH were in Tanner stages I/II at baseline. Bone density accrual (Δ) adjusted for age, Tanner stage and baseline DXA *Z*-score was less in boy CWH than boy CWOH {adjusted mean (95% confidence interval (CI)] ΔLS-BMAD *Z*-score −0.14 (−0.25 to −0.02) vs. 0.01 (−0.09 to 0.12), *P* = 0.020, and ΔTBLH-BMC^LBM^*Z*-score −0.19 (−0.33 to −0.04) vs. 0.07 (−0.07 to 0.20), *P* = 0.015}, but similar in girls with and without HIV [ΔLS-BMAD *Z*-score 0.05 (−0.07 to 0.17) vs. −0.01 (−0.09 to 0.07), *P* = 0.416, and ΔTBLH-BMC^LBM^*Z*-score 0.08 (−0.07 to 0.22) vs. −0.03 (−0.12 to 0.07), *P* = 0.295]. Viral load greater than 1000 copies/ml and tenofovir disoproxil fumarate use were associated with less gain in LS-BMAD *Z*-score among boys, whereas Tanner stage IV and V were associated with greater gains in LS-BMAD and TBLH-BMC^LBM^*Z*-scores among CWH.

**Conclusion:**

Among boys only, CWH had impaired bone accrual, associated with high viral load and tenofovir use. Bone density gains were greater in later puberty among CWH suggesting potential to correct deficits.

## Introduction

The growing paediatric population living with HIV experience chronic HIV-associated morbidities as they transition through adolescence, including low bone density, which increases risk of fractures and the development of osteoporosis in later adulthood [1–3]. Puberty is an important period when rapid linear growth and bone mass accrual occur until peak bone mass is reached in early adulthood [4–6]. The rate of pubertal development differs by sex, with girls having an earlier onset and shorter duration of puberty compared to boys [7–9].

Stunting (height-for-age *Z*-score <−2) is common among African children with HIV (CWH) [10]. Furthermore, a higher prevalence of low bone density is observed among prepubertal and pubertal CWH compared to children without HIV infection (CWOH) [11,12], and to children who are HIV-exposed but uninfected [13]. In a cross-sectional analysis of CWH established on antiretroviral therapy (ART) aged 8–16 years in Zimbabwe, we identified marked deficits in skeletal size-adjusted bone density among CWH compared to CWOH that were more pronounced in those at the end of puberty and were associated with stunting [14]. There are few studies describing bone density accrual in children growing up with perinatal HIV. A 2-year longitudinal cohort study conducted among South African prepubertal CWH showed persistently lower bone mass compared to CWOH at both baseline and follow-up visits, but no difference in the annual percentage change of bone mass between groups [15].

Since the effect of living with HIV on peripubertal bone growth is unclear, we aimed to investigate bone density accrual over 1 year among peripubertal CWH compared to CWOH. We further aimed to identify factors associated with bone density accrual among CWH.

## Methods

### Study design and participants

The IMVASK (Impact of vertical HIV infection on child and adolescent skeletal development) cohort study recruited CWH aged 8–16 years, established on ART for at least 2 years, from HIV clinics at the two public hospitals, Parirenyatwa and Sally Mugabe Central Hospitals, in Harare, Zimbabwe. CWOH in the same age range were recruited from government schools in the corresponding catchment areas using quota sampling by sex and age group (8–10, 11–13 and 14–16 years) to ensure equal numbers per group (Supplementary Table 1) [16]. Exclusion criteria included children requiring immediate hospitalisation, residing outside Harare, or unaware of their HIV diagnosis. Baseline recruitment occurred between June 2018 and November 2019 with a follow-up clinic between May 2019 and February 2021. The first National lockdown in Zimbabwe due to the COVID-19 pandemic started on 30 March 2020 resulting in delayed follow-up for some participants. Follow-up visits were completed for 47.2% prior to this date and study activity resumed from May 2020.

### Questionnaire

At both baseline and follow-up visits, research staff administered questionnaires to collect sociodemographic (age, sex, orphanhood), lifestyle (physical activity, vitamin D and calcium intake, history of tuberculosis) and HIV-specific data [age at diagnosis, age at ART initiation, ART regimens and duration of ART (calculated as a percentage of years lived)].

Physical activity was assessed using the International Physical Activity Questionnaire (IPAQ), validated in multiple countries including South Africa [17], and quantified as multiples of the resting metabolic rate in MET-minutes (metabolic equivalent of task). Vitamin D and calcium intake were assessed using a questionnaire-based tool validated in India and Malawi and adapted to Zimbabwe in accordance with international guidelines [16,18]. The tool estimates dietary intake based on the number of locally relevant animal-sourced food types (eggs, dairy, fish, meat, fortified oils, margarine and kapenta fish) consumed at least three times per week.

### Clinical examination

Two standing height and weight measurements were taken to the nearest 0.1 cm (using a Seca 213 stadiometer) and 0.1 kg (using Seca 875 weight scales) respectively. If height measurements differed by more than 0.5 cm, or weight measurements by more than 0.5 kg, a third reading was taken, and the final height and weight values was the mean of the two or three measurements. Height-for-age and weight-for-age *Z*-scores were calculated using 1990 UK reference data [19]. Tanner pubertal staging was assessed by a nurse or doctor. Testicular volume, penile size (length and circumference) and pubic hair growth (quality, distribution and length) were assessed for boys, while breast size and contour, pubic hair growth and age of menarche were assessed for girls. These characteristics were used to grade the children from I to V based on Tanner descriptions [20–22]. Where there was discordance between the stages, testicular and breast development stage were used to assign Tanner stage for boys and girls, respectively.

### Bone density measurement

Trained radiographers performed dual X-ray absorptiometry (DXA) scans using a Hologic QDR WI densitometer with Apex software V.4.5 (Hologic, Bedford, Massachusetts, USA). Repeat scans were performed in a subgroup of participants (*n* = 30) to confirm reproducibility. Bone measurements included total-body less-head bone mineral content for lean body mass (TBLH-BMC^LBM^) and lumbar spine bone mineral apparent density (LS-BMAD). As per the International Society of Clinical Densitometry (ISCD) recommendations, LS-BMAD was calculated from DXA-measured lumbar spine data using the Carter method [23]. TBLH-BMC^LBM^ was calculated using published equations for Hologic DXA scans that adjust for log-transformed total body lean mass, total body fat mass, height and age [24]. Measurements were adjusted for skeletal size, as per ISCD recommendations, as the two-dimensional bone density are highly dependent on bone size; thus, DXA underestimates bone density in children [25]. Bone density *Z*-scores were generated using a Hologic UK population reference dataset of white children aged 4–20 years, collected from 1996 to 2012 as recommended by the ISCD, as no local reference data were available [24,26].

### Blood sample collection

Children who were not known to have HIV infection on enrolment underwent a rapid HIV test as part of their assessment. One participant tested positive and was referred for HIV care and excluded from this study (Supplementary Table 1). In children with a known HIV diagnosis on enrolment, CD4^+^ T-cell count, and HIV viral load were measured using an Alere PIMA CD4 machine (Waltham, Massachusetts, USA) and the GeneXpert platform (Cepheid, California, USA) respectively. Viral suppression was defined as 50 copies/ml or less.

### Statistical analysis

Analyses were performed using RStudio (version 4.1.2).

A socioeconomic status (SES) score was derived using principal component analysis that combined a list of variables (number of people in the household, age of the head of the household, maternal and paternal education, ownership of the household, monthly income, access to electricity, water and flush toilet, and household ownership of fridge, bicycle, car, television or radio) split into tertiles.

The dataset had 93.8% complete entries, with the ‘orphanhood’ variable having the most missingness at 2.4%. Missing data were imputed using multiple imputation by chained equations, and assuming data were missing at random [27]. Imputed variables included bone density outcomes and previously mentioned participant characteristics including age, sex, Tanner stage, SES, orphanhood, physical activity, dietary calcium and vitamin D intakes, and history of tuberculosis. Sociodemographic characteristics of participants who completed follow-up visit were compared to those lost-to-follow-up.

Data were sex-stratified given known sex-specific differences in pubertal maturation [9] and compared between CWH and CWOH using independent sample *t* tests for means and chi-squared (*χ*^2^) tests for proportions. Annualised change (Δ) in LS-BMAD, TBLH-BMC^LBM^ and height *Z*-scores were calculated as the difference between baseline and follow-up measurements, divided by the number of days between visits, multiplied by 365.25 days (this accounted for variation in follow-up period). Means and mean differences with 95% confidence intervals (CIs) for ΔLS-BMAD, ΔTBLH-BMC^LBM^ and Δheight *Z*-scores between CWH and CWOH were calculated using linear regression adjusting for baseline age, Tanner stage and bone and height measurements [14,15,28]. Interactions between HIV status, age and Tanner stage on change in bone densities and height were investigated. To visualize growth patterns, a locally estimated scatterplot smoothing method (LOESS) was used to plot ΔLS-BMAD, ΔTBLH-BMC^LBM^ and Δheight *Z*-scores against age.

Among the CWH who completed follow-up, associations between HIV-related factors and ΔLS-BMAD and ΔTBLH-BMC^LBM^*Z*-scores, stratified by sex, were investigated using a linear regression model adjusted for first visit (baseline) covariates (determined a priori) including age, Tanner stage and baseline *Z*-score. Potential confounding variables were identified and assessed using a directed acyclic graph to inform the necessary adjustments. HIV-related factors included baseline ART duration (years), HIV viral load (≤50, 50–1000, ≥1000 copies/ml) and current use of tenofovir disoproxil fumarate (TDF). Physical activity level at baseline (low <600 MET minutes/week, moderate 600–3000 MET minutes/week, high >3000 MET minutes/week) was also investigated given its potential skeletal benefits [29,30].

### Ethical considerations

Written informed consent was obtained from parents or guardians and assent from children. The study was approved by the Medical Research Council of Zimbabwe (MRCZ/A/2297), Parirenyatwa Hospital and College of Health Sciences Joint Research Ethics Committee (JREC/11/18), the Harare Central Hospital Ethics Committee (HCHEC 170118/04), the Biomedical Research and Training Institute Institutional Review Board (AP145/2018) and the London School of Hygiene and Tropical Medicine (15333) Ethics Committee.

## Results

### Study population

Of 609 participants recruited, 492 (80.8%) completed the 1-year follow-up visit (Supplementary Figure 1). Characteristics of those who did and did not complete a follow-up visit were largely similar except for being older and girls having higher daily dietary calcium intakes (Supplementary Table 1). Characteristics between participants with complete data and those missing at least one bone outcome at follow-up visit were similar except for daily dietary calcium and vitamin D intake among girls (Supplementary Table 3). There were no differences between participants with complete data for bone density outcomes, Tanner stage, orphanhood and CD4^+^ T-cell count and viral load (for CWH), compared to those missing one or more variable (Supplementary Table 4).

Follow-up duration was mean ± SD 445 ± 95 days overall (CWH: 475 ± 104 days and CWOH: 415 ± 73 days). The cross-sectional demographic characteristics and bone and height baseline data presented are of participants who completed follow-up.

Among the 492 participants who completed a follow-up visit, those with and without HIV were of similar ages, whilst those with HIV were more likely to have been orphaned, have a history of tuberculosis, and to have reported a lower physical activity level at their baseline visit (Table 1). Having HIV was weakly associated with lower SES among girls. Daily dietary calcium and vitamin D intakes did not differ by HIV status or sex. At both baseline and follow-up visits, more girls with HIV were in earlier Tanner stages compared to those without HIV; for boys, there was no association of HIV status with Tanner stage (Table 1 and Supplementary Table 2).

**Table 1:** Baseline characteristics of study participants who were followed up to 1 year.

	Boys N = 247			Girls N = 245		
	HIV Negative *n* = 122	HIV Positive *n* = 125	*p*-value	HIV Negative *n* = 126	HIV Positive *n* = 119	*p*-value
Sociodemographic characteristics						
Age years, mean (SD)	12.4 (2.5)	12.6 (2.5)	0.383	12.7 (2.6)	12.4 (2.5)	0.385
Socioeconomic status, n(%)						
Tertile 1 (low)	30 (24.6)	45 (36.0)		44 (34.9)	49 (41.2)	
Tertile 2 (middle)	44 (36.1)	39 (31.2)		34 (27.0)	41 (34.5)	
Tertile 3 (high)	48 (39.3)	41 (32.8)	0.148	48 (38.1)	29 (24.4)	0.067
Orphanhood (one or both parents deceased)^a^	9 (7.4)	51 (40.8)	<0.001	9 (7.1)	52 (43.7)	<0.001
Pubertal stage						
Tanner I	37 (30.3)	49 (39.2)		23 (18.3)	48 (40.3)	
Tanner II	32 (26.2)	31 (24.8)		26 (20.6)	19 (16.0)	
Tanner III	19 (15.6)	16 (12.8)		23 (18.3)	25 (21.0)	
Tanner IV	31 (25.4)	17 (13.6)		39 (31.0)	17 (14.3)	
Tanner V	2 (1.6)	4 (3.2)	0.157	14 (11.1)	5 (4.2)	<0.001
Lifestyle factors						
Physical activity level						
Low, <600 MET mins/week	41 (33.6)	58 (46.4)		49 (38.9)	63 (52.9)	
Moderate, 600–3000 MET mins/week	43 (35.2)	28 (22.4)		34 (27.0)	29 (24.4)	
High, >3000 MET mins/week	38 (31.1)	39 (31.2)	0.048	43 (34.1)	27 (22.7)	0.061
Daily dietary calcium intake						
Very low, <150 mg/day	54 (44.3)	53 (42.4)		53 (42.1)	46 (38.7)	
Low, 150–299 mg/day	24 (19.7)	24 (19.2)		28 (22.2)	29 (24.4)	
Moderate, 300–450 mg/day	44 (36.1)	48 (38.4)	0.929	45 (35.7)	44 (37.0)	0.850
Daily dietary vitamin D						
Very low, <4.0 μg/day	13 (10.7)	21 (16.8)		18 (14.3)	13 (10.9)	
Low, 4.0–5.99 μg/day	79 (64.8)	82 (65.6)		81 (64.3)	81 (68.1)	
Moderate, 6.0–8.0 μg/day	30 (24.6)	22 (17.6)	0.209	27 (21.4)	81 (68.1)	0.710
Past or current tuberculosis	1 (0.8)	21 (16.8)	<0.001	0 (0.0)	16 (13.4)	<0.001
HIV characteristics						
Age at HIV diagnosis years, median(IQR)	–	3.0 (1.2; 5.8)	–	–	3.0 (1.2; 5.9)	–
Age at ART initiation years, median (IQR)	–	3.8 (2.0; 6.8)	–	–	3.7 (1.8; 7.5)	–
% of life on ART, mean (SD)	–	64.7 (22.3)	–	–	65.0 (22.9)	–
TDF use (at baseline visit)^b^, n(%)	–	40 (33.0)	–	–	38 (32.2)	–
Viral load^c^, n(%)						
≤50 copies/ml	–	61 (51.7)	–	–	65 (57.5)	–
50–1000 copies/ml	–	34 (28.8)	–	–	21 (18.6)	–
≥1000 copies/ml	–	23 (19.5)	–	–	27 (23.9)	–
CD4+ T-cell count <500 cells/μL^d^, n(%)	–	27 (22.0)	–	–	21 (19.1)	–
Anthropometry						
Height (cm), mean (SD)	146.7(14.8)	139.7 (12.3)	<0.001	147.1 (12.0)	140.1 (13.3)	<0.001
Height Z-score, mean (SD)	−0.4 (1.2)	−1.7 (1.1)	<0.001	−0.6 (1.2)	−1.6 (1.0)	<0.001
Height-for-age Z-score <-2^e^, n (%)	10 (8.2)	46 (36.8)	<0.001	13 (10.4)	41 (34.7)	<0.001
Weight (kg), mean (SD)	37.6 (11.1)	32.7 (7.6)	<0.001	42.0 (13.0)	35.2 (10.9)	<0.001
Weight-for-age Z-score, mean (SD)	−0.8 (1.0)	−1.8 (1.1)	<0.001	−0.4 (1.2)	−1.4 (1.1)	<0.001
Weight-for-age Z-score <-2^f^, n(%)	15 (12.4)	49 (39.2)	<0.001	9 (7.2)	31 (26.3)	<0.001
Bone density measures						
LS-BMAD (g/cm^3^), mean (SD)	0.193 (0.028)	0.186 (0.030)	0.082	0.227 (0.036)	0.217 (0.034)	0.021
LS-BMAD Z-score, mean (SD)	−0.60 (1.15)	−0.92 (1.41)	0.056	0.18 (1.10)	−0.14 (1.26)	0.034
TBLH-BMC^LBM^ (g), mean (SD)	1006.27 (338.21)	860.32 (253.11)	<0.001	1084.21 (343.67)	919.35 (290)	<0.001
TBLH-BMC^LBM^ Z-score, mean (SD)	−0.57 (0.91)	−0.67 (1.03)	0.393	−0.18 (1.02)	−0.59 (1.2)	0.004

MET, resting metabolic rate; TDF, tenofovir disoproxil fumarate; LS-BMAD, lumbar spine bone mineral apparent density; TBLH-BMC^LBM^, total body- less head bone mineral content for lean body mass. Missing datapoints:

a Orphanhood (one boy without HIV, six boys with HIV; two girls without HIV; four girls with HIV). Tanner stage (one boy without HIV, eight boys with HIV; one girl without HIV, five girls with HIV).

b Missing ART regimen (five boys, one girl).

c Viral load <50 copies/ml (seven boys; six girls).

d CD4^+^ cell count <500 cell/μl (two boys; nine girls).

e Defined as stunted growth.

f Defined as underweight.

Among CWH who were successfully followed-up, median age at HIV diagnosis, age at ART initiation, duration of ART as a percentage of years lived, proportion on a TDF-based regimen, viral load less than 50 copies/ml, and CD4^+^ T-cell count greater than 500 cells/μl were similar between sexes (Table 1). There were 50 children who were less than 10 years old, of whom 3 (all boys) were taking tenofovir at baseline. Of the 193 children older than 10, 75 (38.9%) were on a TDF-based regimen. Other reported ART regimens at baseline included Zidovudine-based and Abacavir-based regimens (Supplementary Table 5).

### Bone density at baseline and follow-up

**Fig. 1:**
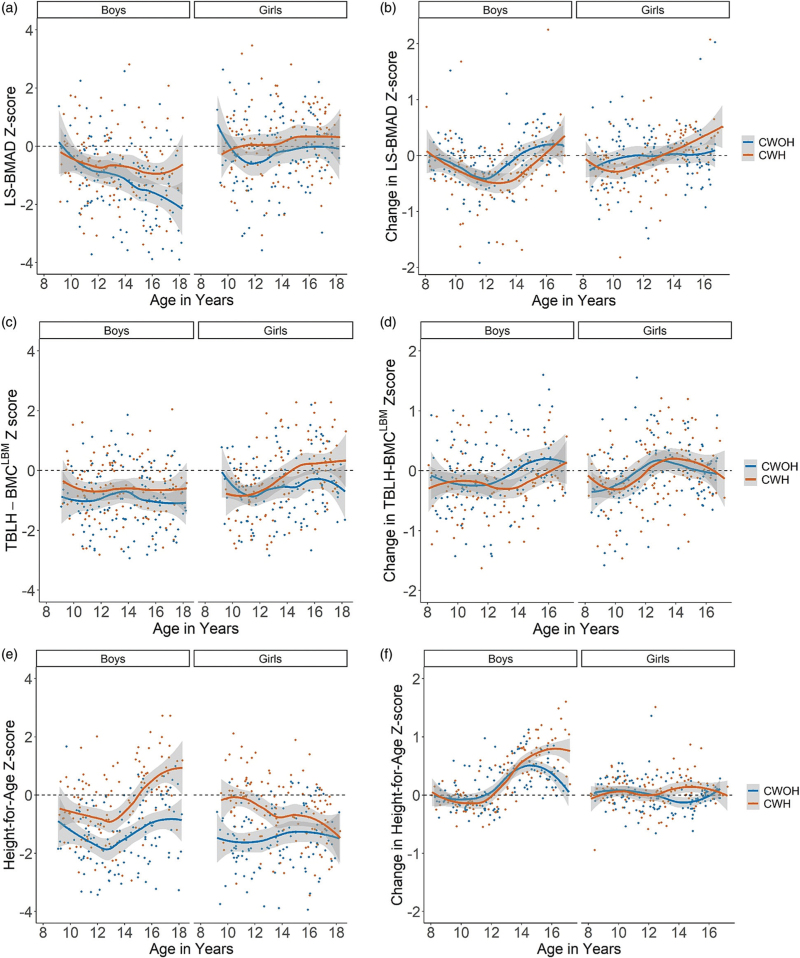
Scatter plot showing age and bone and height *Z*-score outcomes. (a) Height-for-age *Z*-score at follow-up; (b) LS-BMAD *Z*-score at follow-up; (c) TBLH-BMC *Z*-score at follow-up; (d) change in height-for-age *Z*-score; (e) change in LS-BMAD *Z*-score and (f) change TBLH-BMC^LBM^*Z*-score, stratified by sex and HIV status (*N* = 492 for all plots).

Of the boys who completed follow-up, absolute TBLH-BMC^LBM^ at baseline was substantially lower among boys with HIV compared to boys without HIV [mean difference (95% CI) 145.95 g (80.91–220.99), *P* < 0.001 (Table 1)]. Weak evidence was observed for a difference in baseline LS-BMAD in boys with and without HIV [0.006 g/cm^3^ (−0.001 to 0.014), *P* = 0.082 (Table 1)]. Although no differences by HIV status were seen in baseline TBLH-BMC^LBM^*Z*-score, weak evidence of a difference was seen in LS-BMAD *Z*-score between boys with and without HIV [0.11 (−0.14 to 0.35), *P* = 0.393 and 0.32 (−0.01 to 0.64), *P* = 0.056 respectively (Table 1)]. At the follow-up visit, absolute and *Z*-score values for LS-BMAD and TBLH-BMC^LBM^ were lower in boys with HIV compared to boys without HIV (Fig. 1a and 1c, Supplementary Table 2).

Age is continuous, and is at follow-up for (a), (c) and (d); and at baseline for (b), (d) and (f). Unadjusted data are shown. Solid line created using cross-validated locally estimated scatterplot smoothing method (span = 0.75). Error bars indicate 95% CIs. Data are presented as grey dots [children living without HIV (CWOH) and black dots (children with HIV (CWH)]. LS-BMAD, lumbar spine bone mineral apparent density. Δ, change in bone or height outcome; TBLH-BMC^LBM^, total body less head bone mineral content for lean body mass.

Girls with HIV who completed follow-up had lower absolute baseline LS-BMAD and TBLH-BMC^LBM^ compared to girls without HIV [mean difference (95% CI) 0.010 g/cm^3^ (0.001–0.019), *P* = 0.021 and 164.86 g (84.66–245.07), *P* < 0.001, respectively (Table 1)]. Baseline LS-BMAD *Z*-scores and TBLH-BMC^LBM^*Z*-scores were also lower among girls with HIV compared to girls without HIV [0.32 (0.02–0.62), *P* = 0.034 and 0.40 (0.13–0.67), *P* = 0.004, respectively (Table 1)]. Similarly, at follow-up visit, LS-BMAD and TBLH-BMC^LBM^ absolute and *Z*-score outcomes were consistently lower in girls with HIV compared to girls without HIV (Supplementary Table 2).

### Annual change in bone density

Both with and without adjustment for age, Tanner stage and baseline bone density, bone density accrual was less in boys with HIV compared to boys without HIV [adjusted mean (95% CI) ΔLS-BMAD *Z*-score −0.12 (−0.23 to −0.01) vs. 0.01 (−0.09 to 0.12) such that the mean difference was −0.14 (−0.25 to −0.02), *P* = 0.020, and adjusted mean ΔTBLH-BMC^LBM^*Z*-score −0.12 (−0.27 to 0.03) vs. 0.07 (−0.07 to 0.20) such that the mean difference was −0.19 (−0.33 to −0.04), *P* = 0.015] (Table 2). This difference was seen most in boys aged 12–16 years (Fig. 1b and 1d). Bone accrual appeared to increase around the age of 12 in boys without HIV, and at the later age of 14 in boys with HIV (Fig. 1b and 1d).

**Table 2:** Mean difference (95% confidence interval) in change in LS-BMAD, TBLH-BMC^LBM^ and height *Z*-scores between participants living with and without HIV.

	Unadjusted *β*^a^ (95% CI)	*P* value	Adjusted β^b^(95% CI)	*P* value
Boys (*n* = 247)				
ΔLS-BMAD *Z*-score^c^	−0.16 (−0.28; −0.04)	0.011	−0.14 (−0.25; −0.02)	0.020
ΔTBLH-BMC^LBM^*Z*-score^c^	−0.16 (−0.31; −0.01)	0.040	−0.19 (−0.33; −0.04)	0.015
ΔHeight *Z*-score^c^	0.12 (−0.02; 0.27)	0.092	0.15 (0.01; 0.28)	0.036
Girls (*n* = 245)				
ΔLS-BMAD *Z*-score^c^	0.02 (−0.10; 0.14)	0.754	0.05 (−0.07; 0.17)	0.416
ΔTBLH-BMC^LBM^*Z*-score^c^	0.02 (−0.12; 0.16)	0.774	0.08 (−0.07; 0.22)	0.295
ΔHeight *Z*-score^c^	0.08 (0.01; 0.14)	0.024	−0.02 (−0.08; 0.05)	0.652

Δ, change in bone or height outcome; LS-BMAD, lumbar spine bone mineral apparent density; TBLH-BMC^LBM^, total body- less head bone mineral content for lean body mass.

a Linear regression model for the mean difference.

b Linear regression model for the mean difference adjusted for baseline age, Tanner stage (included as a variable with five levels) and baseline DXA *Z*-score and checking for interaction terms between HIV status, Tanner stage and age.

c Change in DXA or height *Z*-score calculated by subtracting the follow-up from baseline *Z*-score and dividing by number of days between visits × 365.25.

However, unlike boys, annualized change in LS-BMAD and TBLH-BMC^LBM^*Z*-scores were similar in girls with and without HIV [adjusted mean (95% CI) ΔLS-BMAD *Z*-score 0.04 (−0.06 to 0.13) vs. −0.01 (−0.09 to 0.07) with an adjusted mean difference 0.05 (−0.07 to 0.17), *P* = 0.416], and ΔTBLH-BMC^LBM^*Z*-score 0.05 (−0.07 to 0.16) vs. −0.03 (−0.12 to 0.07), with adjusted mean difference 0.08 (−0.07 to 0.22), *P* = 0.295]. Although bone density at follow-up was lower among older girls living with HIV (Fig. 1a and 1c), bone accrual was similar among girls with and without HIV (Fig. 1b and 1d). No evidence was detected for two-way interactions between variables age, HIV status and Tanner stage (as an ordinal variable) on ΔLS-BMAD or ΔTBLH-BMC^LBM^, in boys or girls.

### Baseline and follow-up height and annual change in height

Among boys who completed follow-up, those living with HIV were shorter than boys without HIV [absolute mean difference (95% CI) at baseline by 7.01 cm (3.61–10), *P* < 0.001 and height-for-age *Z*-score −1.08 (0.83–1.34), *P* < 0.001 (Table 1)]. This difference persisted at follow-up (Fig. 1e and Supplementary Table 2). Boys with HIV gained more height compared to boys without HIV over 1 year [adjusted mean (95% CI) Δheight 0.32 (0.01–0.28) vs. 0.17 (0.05– 0.30) with a mean difference of 0.15 (0.01–0.28), *P* = 0.036]. At follow-up, older boys with HIV were continuing to gain height-for-age *Z*-score, whilst change in height-for-age *Z*-score among boys without HIV was similar to that of the reference population (Fig. 1f).

At both baseline and follow-up, absolute height and height-for-age *Z*-scores were lower among girls with HIV compared to girls without HIV [mean difference (95% CI) 7.00 cm (3.81–10.20), *P* < 0.001 and 0.91 (0.64–1.19), *P* < 0.001 (Table 1, Fig. 1e and Supplementary Table 2). Annual mean height *Z*-score accrual was similar among girls with and without HIV [adjusted mean difference −0.02 (−0.08 to 0.05), *P* = 0.652]. Height gains were similar in girls with and without HIV until age 14 where girls with HIV appear to gain less height compared to their peers (Fig. 1f).

### Factors associated with change in bone density among children with HIV

Boys in later Tanner stages, gained more LS-BMAD *Z* score compared to boys in earlier Tanner stages (Table 3). Among girls, those in Tanner stages II to IV had greater gains in ΔLS-BMAD and ΔTBLH-BMC^LBM^*Z*-scores compared to those in Tanner stage I, with a trend towards greater gains at later Tanner stages (Table 4). Higher baseline *Z*-scores were associated with less gain in bone density among both boys (Table 3) and girls (Table 4).

**Table 3:** Characteristics associated with annualized change in LS-BMAD and TBLH-BMC^LBM^*Z*-scores among boys with HIV.

	Boys							
	ΔLS-BMAD Z-score				ΔTBLH-BMC Z-score			
	Unadjusted *β* Coefficient (95% CI)	*p* value	Adjusted *β* (95% CI)	*p* value	Unadjusted *β* Coefficient (95% CI)	*p* value	Adjusted *β* (95% CI)	*p* value
Age (per year)	0.03 (−0.01; 0.07)	0.122	−0.01 (−0.07 to 0.05)	0.725	0.00 (−0.05; 0.05)	0.963	0.02 (−0.06; 0.09)	0.691
Tanner Stage								
I	–		–		–		–	
II	−0.15 (−0.38; 0.08)	0.015	−0.14 (−0.38; 0.10)	0.012	−0.16 (−0.45; 0.14)	0.254	−0.18 (−0.50; 0.13)	0.689
III	−0.11 (−0.39; 0.18)		−0.11 (−0.45; 0.23)		−0.06 (−0.43 0.31)		−0.10 (−0.54; 0.34)	
IV	0.38 (0.11; 0.65)		0.45 (0.07; 0.82)		−0.04 (−0.39; 0.32)		−0.14 (−0.64; 0.35)	
V	0.42 (−0.11; 0.95)		0.52 (−0.11; 0.95)		0.35 (−0.33; 1.03)		0.28 (−0.55; 1.11)	
Baseline Z-score^a^	−0.12 (−0.19; −0.06)	<0.001	−0.12 (−0.19; −0.05)	0.001	−0.25 (−0.36; −0.15)	<0.001	−0.27 (−0.39; −0.15)	<0.001
Physical Activity^b^								
High	–	0.793	–	0.540	–	0.564	–	0.417
Moderate	−0.13 (−0.40; 0.14)		−0.09 (−0.33; 0.14)		−0.24 (−0.56; 0.08)		−0.15 (−0.47; 0.16)	
Low	−0.03 (−0.25; 0.19)		−0.10 (−0.31; 0.11)		−0.08 (−0.35; 0.19)		−0.16 (−0.43; 0.12)	
ART duration (per year)	0.00 (−0.03; 0.04)	0.906	−0.02 (−0.06; 0.02)	0.362	0.03 (−0.01; 0.07)	0.154	0.03 (−0.02; 0.07)	0.197
Viral Load (copies/ml)								
<50	–	0.256	–	0.073	–	0.896	–	0.054
50–1000	0.10 (−0.12; 0.32)		0.09 (−0.12; 0.29)		−0.10 (−0.38; 0.18)		0.03 (−0.24; 0.31)	
≥1000	−0.06 (−0.31; 0.19)		−0.24 (−0.48; 0.00)		−0.23 (−0.55; 0.09)		−0.28 (−0.60; 0.04)	
TDF Use								
No	–	0.787	–	0.033	–	0.739	–	0.523
Yes	−0.03 (−0.28; 0.21)		−0.28 (−0.19; −0.05)		0.05 (−0.25; 0.35)		−0.11 (−0.44; 0.23)	

Δ, change in bone outcomes; ART, antiretroviral therapy; LS-BMAD, lumbar-spine bone mineral apparent density; TBLH-BMC^LBM^, total body less head bone mineral content for lean body mass; TDF, tenofovir disoproxil fumarate. *β* coefficient denotes change (Δ) bone density outcome for each one-unit change in the predictor variable adjusting for exposure variables including age, Tanner stage, baseline *Z*-score, physical activity, ART duration, HIV viral load, TDF exposure (*N* = 125).

a *Z*-score change per baseline *Z*-score.

b High physical activity was >3000 resting metabolic minutes per week; moderate was 600–3000 resting metabolic minutes per week; low was <600 resting metabolic minutes per week.

**Table 4:** Characteristics associated with annualized change in LS-BMAD and TBLH-BMC^LBM^*Z*-scores among girls with HIV.

	Girls							
	ΔLS-BMAD Z-score				ΔTBLH-BMC Z-score			
	Unadjusted *β* Coefficient (95% CI)	*p* value	Adjusted *β* (95% CI)	*p* value	Unadjusted *β* Coefficient (95% CI)	*p* value	Adjusted *β* (95% CI)	*p* value
Age (per year)	0.09 (0.06; 0.12)	<0.001	0.00 (−0.06; 0.07)	0.906	0.07 (0.03; 0.11)	0.001	−0.03 (−0.11 to 0.04)	0.398
Tanner Stage								
I	–		–		–		–	
II	0.46 (0.24; 0.68)	<0.001	0.41 (0.14; 0.67)	<0.001	0.32 (0.05; 0.60)	0.028	0.38 (0.05; 0.71)	0.016
III	0.51 (0.31; 0.71)		0.52 (0.22; 0.82)		0.54 (0.29; 0.79)		0.70 (0.32; 1.08)	
IV	0.36 (0.14; 0.59)		0.50 (0.14; 0.87)		0.34 (0.05; 0.62)		0.63 (0.18; 1.08)	
V	0.94 (0.55; 1.32)		1.04 (0.50; 1.57)		0.58 (0.09; 1.07)		0.87 (0.20; 1.55)	
Baseline Z-score^a^	−0.12 (−0.18; −0.05)	0.001	−0.10 (−0.16; −0.03)	0.004	−0.14 (−0.23; −0.05)	0.003	−0.12 (−0.21; −0.03)	0.009
Physical Activity^b^								
High	–		–		–		–	
Moderate	0.05 (−0.21; 0.31)	0.955	0.03 (−0.20; 0.26)	0.739	−0.12 (−0.42; 0.19)	0.818	−0.14 (−0.43; 0.15)	0.680
Low	0.01 (−0.23; 0.22)		−0.03 (−0.22; 0.16)		0.03 (−0.23; 0.29)		0.06 (−0.19; 0.30)	
ART duration (per year)	−0.01 (−0.05; 0.02)	0.410	−0.03 (−0.06; 0.01)	0.145	−0.02 (0.06; 0.02)	0.412	−0.01 (−0.05; 0.03)	0.624
Viral Load (copies/ml)								
<50	–		–		–		–	
50–1000	0.16 (−0.09; 0.40)	0.809	0.13 (−0.08; 0.34)	0.244	0.02 (−0.26; 0.30)	0.261	−0.00 (−0.27; 0.26)	0.796
>1000	0.03 (−0.20; 0.25)		−0.11 (−0.30; 0.08)		−0.15 (−0.40; 0.11)		−0.18 (−0.43; 0.06)	
TDF Use								
No	–	0.408	–	0.250	–	0.320	–	0.893
Yes	0.09 (−0.12; 0.29)		−0.12 (−0.33; 0.09)		0.12 (−0.12; 0.37)		−0.02 (−0.28; 0.25)	

Δ, change in bone outcomes; ART, antiretroviral therapy; LS-BMAD, lumbar-spine bone mineral apparent density; TBLH-BMC^LBM^, total body less head bone mineral content for lean body mass; TDF, tenofovir disoproxil fumarate. *β* coefficient, change (Δ) bone density outcome for each one-unit change in the exposure variable adjusting for exposure variables including age, Tanner stage, baseline *Z*-score, physical activity, ART duration, HIV viral load, TDF exposure (*N* = 119).

a *Z*-score change per baseline *Z*-score.

b High physical activity was >3000 resting metabolic minutes per week; moderate was 600–3000 resting metabolic minutes per week; low was <600 resting metabolic minutes per week.

Among HIV-specific factors, viral load of at least 1000 copies/ml was associated with less gain in bone density among boys [adjusted ΔLS-BMAD *Z*-score −0.24 (−0.48 to 0.00) *P* = 0.073 and ΔTBLH-BMC^LBM^*Z*-score −0.28 (−0.60 to 0.04), *P* = 0.054 (Table 3)). Use of TDF-containing ART was associated with less gain in LS-BMAD *Z*-scores among boys [−0.28 (−0.19 to −0.05), *P* = 0.033 (Table 3)]; however, this was not observed among girls. Age, physical activity and duration of ART were not associated with ΔLS-BMAD or ΔTBLH-BMC^LBM^*Z*-score in neither boys nor girls (Tables 3 and 4).

## Discussion

We have shown that, over 1 year of childhood growth, girls with and without HIV had similar gains in bone density, while boys with HIV gain less bone density than boys without HIV. Later Tanner stage was associated with gains in LS-BMAD and TBLH-BMC^LBM^, suggesting that the deficits seen in earlier Tanner stages may be partially recovered as CWH progress through an albeit later puberty. Among HIV-relevant factors, high viral load and use of TDF-based ART were associated with attenuated bone density gains in boys.

Few data quantify changes in bone density among adolescents from highly HIV endemic regions, and fewer still investigate sex-specific effects. In the United States, Jacobson *et al.*[28] showed that lower total body bone mineral content and total body and spinal bone mineral density were more pronounced at later Tanner stages (V vs. III–IV) among boy but not girl CWH, compared to CWOH (mean age 12.6 and 11.9 years, respectively). However, this study was cross-sectional, with no size-adjustment made for DXA-measured bone density. In a cross-sectional study of Thai and Indonesian adolescents (mean age 15 years), low LS-BMAD *Z*-scores were seen in older girls, suggesting bone deficits are more pronounced towards the end of puberty [31]. As HIV can delay the onset of puberty, gains in height and bone mass may also be delayed [32]. This could explain the positive trend between later Tanner stage and LS-BMAD accrual in boys and LS-BMAD and TBLH-BMC^LBM^ accrual in girls. Physiologically, girls start puberty earlier than boys [7]. As there were more boys at earlier Tanner stages (I and II) in our study, this could explain the attenuated gains in bone density observed overall in boys, not seen in girls. In Zimbabwe, adult women with HIV have been shown to have lower bone density than HIV-negative women; however, similar data from adult men with HIV, or between sex differences are currently lacking [33].

Sex-specific mechanisms compromising bone accrual among CWH are likely to be multifactorial. Sex hormone differences may affect patterns of bone accrual [34]. Among girls, oestrogen increases endocortical thickness and inhibits periosteal bone formation, while among boys, androgens favour periosteal apposition and cortical thickness [35]. This results in men usually having larger, denser bones than women. A meta-analysis by Santi *et al.*[36] reported higher prevalence of hypogonadism and low testosterone among men with HIV [mean age (±SD) 42.4 ± 6.2 years] compared to men without HIV, attributed to both direct HIV effects and as a side effect of ART. For the IMVASK study, the two bone outcomes together reflect overall bone accrual; LS-BMAD measures largely trabecular bone (consisting of lacunae and osteocytes in a lattice-like network, giving bone its lightness), whilst TBLH-BMC^LBM^ represents predominantly cortical bone (consisting of lacunae and osteocytes arranged in concentric circles, giving bone its strength). Our finding that boys with HIV have less gain in both bone compartments may suggest an overall skeletal effect of HIV on anabolic hormones, particularly androgens, either directly or through indirect effects of impaired nutrition and/or intercurrent infections associated with HIV [8]. During the pre-ART era, HIV was frequently linked to growth hormone resistance and reduced insulin-like growth factor-1 (IGF-1), both of which are known to impede bone growth [37]. Even with ART and viral control, individuals with HIV often continue to exhibit low levels of growth hormone and IGF-1. This persistence may be due to chronic inflammation, which is believed to suppress hormone production [38].

Approximately 20% of boys and girls with HIV had a viral load of at least 1000 copies/ml. High HIV viral load at baseline among boys with HIV was associated with less gain in LS-BMAD and TBLH-BMC^LBM^*Z*-score. Use of TDF-based ART was associated with less LS-BMAD gain. In Zimbabwe, TDF is part of the first-line ART regimen in both adults and in children when they reach 10 years of age and weigh above 30 kg [39]. In our previously published cross-sectional IMVASK analysis, we found a strong negative association between TDF exposure and both bone outcomes [14]. Schtscherbyna *et al.*[40] also showed lower lumbar spine and total body bone mineral density among Brazilian adolescents with perinatally acquired HIV (mean age 17.3 years) on TDF-based regimens. The indirect effect of TDF on renal phosphate handling is implicated as a potential mechanism behind bone loss observed in adults [41]. Newer agents such as tenofovir alafenamide may be promising for reducing bone-related morbidity due to its lower impact on bone health and kidney function compared to TDF-based regimens [42].

At baseline, lower levels of physical activity were reported in both boys and girls with HIV, compared to children without HIV. Furthermore, most CWH had low (and lower than recommended) levels of physical activity [43]. These patterns have been seen before among Mozambican adolescents with HIV [44]. Potential explanations include both physiological and social factors, such as disease-related fatigue, intercurrent infection, and HIV-associated stigma [44,45]. However, physical activity levels were not associated with change in LS-BMAD or TBLH-BMC^BMC^ outcome in our analysis. It is likely that any effect of physical activity on bone density is only observed over longer periods of time than were studied here.

The strengths of this study are that it is a well powered, longitudinal analysis with minimal loss to follow-up, that compared children with HIV to a population representative sample of children without HIV. Although DXA measures were determined according to ISCD recommendations with *Z*-scores generated [46], DXA-scan measurements underestimate areal bone density in small skeletons (as occurs with HIV), and the reference data used were from British children who were mainly white. Unfortunately, to our knowledge, no equivalent reference data exist locally or across Africa. We are unable to conclude whether this partial recovery will fully resolve bone deficits by the end of puberty, as peak bone mass had not been reached. Ideally, adolescents would be followed up again at the end of puberty for further measurement. We recognize that residual confounding may persist due to past or current biological or environmental factors that were not considered in the initial IMVASK protocol [16].

In conclusion, boys with HIV have impaired bone density accrual through puberty, and this is worse in those with high viral load and who use TDF. Bone density gains in later puberty, in both boys and girls with HIV, suggest that there may be potential to correct or partially correct deficits. This study thus highlights the importance of effective treatment as well as the need to investigate bone accrual later into young adulthood, to inform strategies to improve bone health outcomes for young adults with HIV.

## Acknowledgements

Contributors: R.R., R.A.F., and C.L.G. conceived the study. R.A.F., C.L.G., R.R., and A.M.R. designed the study protocol. R.R. and C.M.-K. collected the data. V.S., R.R., L.J., T.B., and A.M.R. were responsible for data management and data verification. L.J., C.L.G., A.Y.Y.H., V.S., R.A.F., A.M.R., S.R.J. analysed the data. L.J. wrote the first draft. All authors contributed to the report. All authors had access to the study data and had final responsibility for the decision to submit for publication.

Funding: L.J. is funded by the Rhodes Trust. R.R. (ref: 206764/Z/17/Z) and R.A.F. (ref: 206316/Z/17/Z) are funded by the Wellcome Trust. C.L.G. by the National Institute of Health Research (ref: NIHR302394). A.Y.Y.H. is funded by the Canadian Institutes of Health Research (ref: 202012HIV-464257–268748); the Chinese Academy of Medical Sciences (CAMS) Innovation Fund for Medical Science (CIFMS), China (ref: 2018-I2M-2-002); and the Thrasher Research Fund (ref: 01662). C.M.-K. was funded by a National Institute of Health Fogarty Trent Fellowship (grant number 2D43TW009539-06). A.M.R. was partially supported by the UK Medical Research Council (MRC) and the UK Department for International Development (DFID) under the MRC/DFID Concordat agreement, which is also part of the European and Developing Countries Clinical Trials Partnership 2 programme supported by the EU (grant number MR/R010161/1). All other authors declare no competing interests. Funders had no role in study methods, analyses or preparation and submission of this manuscript.

### Conflicts of interest

There are no conflicts of interest.

## Supplementary Material

Supplemental Digital Content
