# Supplementary material for: Reduced bone density accrual among peripubertal boys with HIV in Zimbabwe
Source: AIDS. 2025 Feb 27;39(6):683–94. doi: 10.1097/QAD.0000000000004134 (PMC11970606; doi:10.1097/QAD.0000000000004134)
Supplement: Supplemental Digital Content [file aids-39-683-s001.docx]

**Supplementary materials**

Participants aged 8 to 16 years old randomly invited to participate

**N=1197**

Government sector HIV clinics

**n=631**

Government schools nearby

**n=536**

- Not living in Harare (n=72)
- ART<2 years (n=52)
- Unaware of HIV status (n=20)
- Acute illness (n=1)

A

- Not living in Harare (n=15)
- Established HIV infection on ART >2 years (n=4)
- <8 or >16 years (n=17)

Eligible children without HIV (CWOH) **(n=500)**

Eligible CWH **(n=486)**

**+ 4** identified from schools

CWH consented **(n=305)**

CWOH consented **(n=307)**

Withdrawn after consent (n=2)

**Enrolled CWH (n=303)**

**Enrolled CWOH (n=306)**

Withdrawn due to new HIV diagnosis (n=1)

- Guardian +/- child refusal (n=98)
- No guardian present (n=87)

- Letter not returned (n=151)
- No guardian present (n=87)

**Follow-up visit CWH (n= 251)**

**Follow-up visit CWOH (n= 241)**

Unable to be contacted for follow-up (n= 52)

Unable to be contacted for follow-up (n= 65)

**Supplementary Figure 1: Study participants recruitment and follow-up after one year**

ART=antiretroviral therapy. CWH= Children with HIV, CWOH= Children without HIV, DXA=dual-energy X-ray absorptiometry.

**Supplementary Table 1: Characteristics of participants at baseline visit who were lost to follow-up compared to those who successfully completed the follow-up visit**

|  | | **Boys**  **N=303 at baseline visit** | | | **Girls**  **N= 306 at baseline visit** | | |
| --- | --- | --- | --- | --- | --- | --- | --- |
|  |  | **Followed-up**  **n=247** | **Lost to follow-up**  **n=56** | **p-value** | **Followed-up**  **n=245** | **Lost to follow-up**  **n=61** | **p-value** |
|  | Living with HIV, n(%) | 125 (50.6) | 27 (48.2) | 0.861 | 119 (48.6) | 32 (52.5) | 0.689 |
| **Socio-demographic characteristics** | Age years, mean (SD) | 12.5 (2.5) | 13.3 (2.6) | 0.065 | 12.6 (2.5) | 13.4 (2.5) | 0.058 |
|  | Socioeconomic status, n(%) |  |  |  |  |  |  |
|  | Tertile 1 (low) | 75 (30.4) | 17 (30.4) | 0.655 | 93 (38.0) | 18 (29.5) | 0.415 |
|  | Tertile 2 (middle) | 83 (33.6) | 22 (29.3) |  | 75 (30.6) | 23 (37.7) |  |
|  | Tertile 3 (high) | 89 (36.0) | 17 (30.4) |  | 77 (31.4) | 20 (32.8) |  |
|  | Orphanhood (one or both parents deceased^*^) | 60 (24.3) | 11 (19.6) | 0.588 | 229 (93.5) | 55 (90.2) | 0.999 |
| **Pubertal Stage^** | Tanner I | 86 (34.8) | 16 (28.6) | 0.337 | 71 (29.0) | 14 (23.0) | 0.738 |
|  | Tanner II | 63 (25.5) | 10 (17.9) |  | 45 (18.4) | 10 (16.4) |  |
|  | Tanner III | 35 (14.2) | 11 (19.6) |  | 48 (19.6) | 14 (23.0) |  |
|  | Tanner IV | 48 (19.4) | 14 (25.0) |  | 56 (22.9) | 18 (29.5) |  |
|  | Tanner V | 6 (2.4) | 3 (5.4) |  | 19 (7.8) | 4 (6.6) |  |
| **Lifestyle factors** | Physical activity level |  |  |  |  |  |  |
|  | Low, <600 MET mins/week | 99 (40.1) | 23 (41.1) | 0.840 | 112 (45.7) | 28 (45.9) | 0.913 |
|  | Moderate, 600-3000 MET mins/week | 71 (28.7) | 14 (25.0) |  | 63 (25.7) | 17 (27.9) |  |
|  | High, >3000 MET mins/week | 77 (31.2) | 19 (33.9) |  | 70 (28.6) | 16 (26.2) |  |
|  | Daily dietary calcium intake |  |  |  |  |  |  |
|  | Very low, <150 mg/day | 107 (43.3) | 29 (51.8) | 0.134 | 99 (40.4) | 36 (59.0) | 0.031 |
|  | Low, 150-299 mg/day | 48 (19.4) | 14 (25.0) |  | 57 (23.3) | 9 (14.8) |  |
|  | Moderate, 300–450 mg/day | 92 (37.2) | 13 (23.2) |  | 89 (36.3) | 16 (26.2) |  |
|  | Daily dietary vitamin D |  |  |  |  |  |  |
|  | Very low, <4.0 μg/day | 34 (13.8) | 8 (14.3) | 0.971 | 31 (12.7) | 4 (6.6) | 0.388 |
|  | Low, 4.0-5.99 μg/day | 161 (65.2) | 37 (66.1) |  | 162 (66.1) | 42 (68.9) |  |
|  | Moderate, 6.0-8.0 μg/day | 52 (21.1) | 11 (19.6) |  | 52 (21.2) | 15 (24.6) |  |
|  | Past or current tuberculosis^**^ | 22 (8.9) | 9 (16.1) | 0.176 | 16 (6.5) | 4 (6.6) | 0.999 |

MET= resting metabolic rate; CWH= children living with HIV; CWOH= children living without HIV

Missing data

^Pubertal stage: Nine boys that were followed-up had missing data; two boys that were lost-to-follow-up (LTFU) had no baseline pubertal stage recorded. Six girls that were followed-up had missing data; one girl that was LTFU had no baseline pubertal stage recorded

*Orphanhood: Seven boys that were followed-up had missing data; two boys that was LTFU had no baseline pubertal stage recorded. Seven girls that were followed-up had missing data; one girl that was LTFU had no baseline orphanhood status recorded.

**Previous or current tuberculosis: two girls that was LTFU had no baseline tuberculosis history recorded

**Supplementary Table 2: Characteristics of study participants at the follow-up visit**

|  | | | **Boys**  **N=247** | | | | | **Girls**  **N= 245** | | | | | | |
| --- | --- | --- | --- | --- | --- | --- | --- | --- | --- | --- | --- | --- | --- | --- |
|  |  |  | **HIV Negative**  **n=122** | | **HIV Positive**  **n=125** | **p-value** | | **HIV Negative**  **n=126** | | | **HIV Positive**  **n=119** | **p-value** | | |
| **Socio-demographic characteristics** | | Age years, mean (SD) | 13.4 (2.5) | | 13.8 (2.5) | 0.176 | | 13.8 (2.6) | | | 13.6 (2.5) | 0.569 | | |
|  |  | Socioeconomic status, n(%) |  | |  |  | |  | | |  |  | | |
|  |  | Tertile 1 (low) | 29 (23.8) | | 52 (41.6) | 0.004 | | 35 (27.8) | | | 49 (41.2) | 0.021 | | |
|  |  | Tertile 2 (middle) | 42 (34.4) | | 41 (32.8) |  |  | 41 (32.5) | | | 41 (34.5) |  |  |  |
|  |  | Tertile 3 (high) | 51 (41.8) | | 32 (25.6) |  |  | 50 (39.7) | | | 29 (24.4) |  |  |  |
|  |  | Orphanhood (one or both parents deceased)* | 10 (8.2) | | 52 (41.6) | <0.001 | | 10 (7.9) | | | 55 (46.2) | <0.001 | | |
| **Pubertal stage** | | Tanner I | 4 (3.3) | | 11 (8.8) | 0.474 | | 9 (7.1) | | | 20 (16.8) | 0.011 | | |
|  |  | Tanner II | 52 (42.6) | | 46 (36.8) |  |  | 20 (15.9) | | | 20 (16.8) |  |  |  |
|  |  | Tanner III | 21 (17.2) | | 25 (20.0) |  |  | 24 (19.0) | | | 25 (21.0) |  |  |  |
|  |  | Tanner IV | 42 (34.4) | | 39 (31.2) |  |  | 41 (32.5) | | | 41 (34.5) |  |  |  |
|  |  | Tanner V | 3 (2.5) | | 4 (3.2) |  |  | 32 (25.4) | | | 13 (10.9) |  |  |  |
| **Lifestyle factors** | | Physical activity level |  | |  |  | |  | | |  |  | | |
|  |  | Low, <600 MET mins/week | 42 (34.4) | | 43 (34.4) | 0.997 | | 79 (62.7) | | | 54 (45.4) | 0.024 | | |
|  |  | Moderate, 600-3000 MET mins/week | 57 (46.7) | | 58 (46.4) |  |  | 39 (31.0) | | | 53 (44.5) |  |  |  |
|  |  | High, >3000 MET mins/week | 23 (18.9) | | 24 (19.2) |  |  | 8 (6.3) | | | 12 (10.1) |  |  |  |
|  |  | Daily dietary calcium intake |  | |  |  | |  | | |  |  | | |
|  |  | Very low, <150 mg/day | 65 (53.3) | | 77 (61.6) | 0.294 | | 68 (54.0) | | | 71 (59.7) | 0.306 | | |
|  |  | Low, 150-299 mg/day | 25 (20.5) | | 25 (20.0) |  |  | 25 (19.8) | | | 15 (12.6) |  |  |  |
|  |  | Moderate, 300–450 mg/day | 32 (26.2) | | 23 (18.4) |  |  | 33 (26.2) | | | 33 (27.7) |  |  |  |
|  |  | Daily dietary vitamin D |  | |  |  | |  | | |  |  | | |
|  |  | Very low, <4.0 μg/day | 22 (18.0) | | 35 (28.0) | 0.158 | | 25 (19.8) | | | 34 (28.6) | 0.135 | | |
|  |  | Low, 4.0-5.99 μg/day | 90 (73.8) | | 79 (63.2) |  |  | 87 (69.0) | | | 78 (65.5) |  |  |  |
|  |  | Moderate, 6.0-8.0 μg/day | 10 (8.2) | | 11 (8.8) |  |  | 14 (11.1) | | | 7 (5.9) |  |  |  |
|  | | Past or current tuberculosis | 0 (0.0) | | 14 (11.2) | <0.001 | | 1 (0.8) | | | 11 (9.2) | 0.006 | | |
| **HIV characteristics** | | Age at HIV diagnosis years, median(IQR) | - | | 3.46  (1.64; 6.02) | - | | - | | | 2.91  (1.24; 6.0) | - | | |
|  |  | Age at ART initiation years, median (IQR) | - | | 3.78  (1.96; 6.79) | - | | - | | | 3.69  (1.76; 7.51) | - | | |
|  |  | % of life on ART, mean (SD) | - | | 68.05 (20.44) | - | | - | | | 68.32 (20.93) | - | | |
|  |  | Current TDF use*^, n(%) | - | | 43 (34.4) | - | | - | | | 43 (36.13) | - | | |
|  |  | Viral load <50 copies/ml^**^, n(%) | - | | 76 (63.33) | - | | - | | | 78 (69.64) | - | | |
|  |  | CD4+ T-cell count <500 cells/µL^***^, n(%) | - | | 34 (28.01) | - | | - | | | 29 (25.66) | - | | |
| **Anthropometry^$^** | | Height (cm), mean (SD) | 151. 6 (14.4) | | 146.2 (12.8) | 0.002 | | 151.1 (10.0) | | | 144.9 (12.3) | <0.001 | | |
|  |  | Height Z-score, mean (SD) | -0.3 (1.2) | | -1.3 (1.2) | <0.001 | | -0.6 (1.2) | | | -1.5 (1.0) | <0.001 | | |
|  |  | Height-for-age Z-score <-2^#^, n (%) | 8 (6.7) | | 33 (26.6) | <0.001 | | 15 (12.3) | | | 36 (30.5) | 0.001 | | |
|  |  | Weight (kg), mean (SD) | 41.5 (11.8) | | 36.9 (9.1) | 0.001 | | 46.0 (12.6) | | | 39.4 (11.8) | <0.001 | | |
|  |  | Weight-for-age Z-score, mean (SD) | -0.7 (1.0) | | -1.7 (1.1) | <0.001 | | -0.3 (1.2) | | | 1.2 (1.1) | <0.001 | | |
|  |  | Weight-for-age Z-score <-2^‡^, n(%) | 12 (10.0) | | 42 (34.1) | <0.001 | | 6 (4.9) | | | 28 (23.7) | <0.001 | | |
| **Bone density measures**† | LS-BMAD (g/cm^3^), mean (SD) | | 0.197 (0.032) | 0.189 (0.031) | | 0.033 | | 0.234 (0.037) | 0.225 (0.037) | | | 0.075 |  |  |
|  | LS-BMAD Z-score, mean (SD) | | -0.71  (1.17) | -1.21  (1.36) | | 0.002 | | 0.15 (1.14) | -0.20  (1.23) | | | 0.023 |  |  |
|  | TBLH-BMC^LBM^ (g), mean (SD) | | 1120.41 (397.56) | 961.52 (304.68) | | <0.001 | | 1177.76 (338.53) | 1029.51 (319.15) | | | 0.001 |  |  |
|  | TBLH-BMC^LBM^ Z-score, mean (SD) | | -0.97  (1.03) | -0.64  (0.95) | | 0.010 | | -0.30  (1.14) | -0.60  (1.14) | | | 0.019 |  |  |

MET= resting metabolic rate. TDF= tenofovir disoproxil fumarate LS-BMAD= Lumbar-spine bone mineral apparent density TBLH-BMC^LBM^=total body less head bone mineral content for lean body mass.

^#^Defined as stunted growth ‡Defined as underweight

Missing datapoints:

*Orphanhood (five boys with HIV; seven girls with HIV)

*^Missing ART regimen( 32 boy, 40 girl)

**Viral load <1000 copies/ml (five boys; seven girls)

***CD4 count <500 cell/µl (four boys; six girls)

$Anthropometry: (Height: one girl. Weight: one boy; one girl)

†Bone density: (LS-BMAD: five boys; seven girls. TBLH-BMC^LBM^: four boys; five girls)

**Supplementary Table 3: Characteristics of participants who did and did not have at least one bone outcome (LS-BMAD or TBLH-BMC^LBM^) at the follow-up visit**

|  | | **Boys**  **N=247** | | | **Girls**  **N=245** | | |
| --- | --- | --- | --- | --- | --- | --- | --- |
|  |  | **DXA**  **n=241** | **Missing DXA**  **n=6** | **p-value** | **DXA**  **n=238** | **Missing DXA**  **n=7** | **p-value** |
| **Socio-demographic characteristics** | Age years, mean (SD) | 13.6 (2.5) | 13.1 (2.8) | 0.603 | 13.7 (2.5) | 13.4 (2.4) | 0.786 |
|  | Socioeconomic status, n(%) |  |  |  |  |  |  |
|  | Tertile 1 (low) | 78 (32.4) | 3 (50.0) | 0.211 | 84 (35.3) | 0 (0.0) | 0.140 |
|  | Tertile 2 (middle) | 80 (33.2) | 3 (50.0) |  | 78 (32.8) | 4 (57.1) |  |
|  | Tertile 3 (high) | 83 (34.4) | 0 (0.0) |  | 76 (31.9) | 3 (42.9) |  |
|  | Orphanhood (one or both parents) deceased^*^ | 61 (25.3) | 1 (16.7) | 0.995 | 64 (26.9) | 1 (14.3) | 0.756 |
| **Pubertal stage^** | Tanner I | 15 (6.2) | 0 (0.0) | 0.948 | 28 (11.8) | 0 (0.0) | 0.576 |
|  | Tanner II | 95 (39.4) | 3 (50.0) |  | 39 (16.4) | 2 (28.6) |  |
|  | Tanner III | 45 (18.7) | 1 (16.7) |  | 47 (19.7) | 2 (28.6) |  |
|  | Tanner IV | 79 (32.8) | 2 (33.3) |  | 81 (34.0) | 1 (14.3) |  |
|  | Tanner V | 7 (2.9) | 0 (0.0) |  | 43 (18.1) | 2 (28.6) |  |
| **Lifestyle factors** | Physical activity level |  |  |  |  |  |  |
|  | Low, <600 MET mins/week | 84 (34.9) | 1 (16.7) | 0.573 | 127 (53.4) | 6 (85.7) | 0.114 |
|  | Moderate, 600-3000 MET mins/week | 111 (46.1) | 4 (66.7) |  | 92 (38.7) | 0 (0.0) |  |
|  | High, >3000 MET mins/week | 46 (19.1) | 1 (16.7) |  | 19 (8.0) | 1 (14.3) |  |
|  | Daily dietary calcium intake |  |  |  |  |  |  |
|  | Very low, <150 mg/day | 138 (57.3) | 4 (66.7) | 0.898 | 137 (57.6) | 2 (28.6) | 0.024 |
|  | Low, 150-299 mg/day | 49 (20.3) | 1 (16.7) |  | 40 (16.8) | 0 (0.0)  0 (0.0) |  |
|  | Moderate, 300–450 mg/day | 54 (22.4) | 1 (16.7) |  | 61 (25.6) | 5 (71.4)  5 (71.4) |  |
|  | Daily dietary vitamin D |  |  |  |  |  |  |
|  | Very low, <4.0 μg/day | 56 (23.2) | 1 (16.7) | 0.087 | 59 (24.8) | 0 (0.0) | 0.003 |
|  | Low, 4.0-5.99 μg/day | 166 (68.9) | 3 (50.0) |  | 161 (67.6) | 4 (57.1) |  |
|  | Moderate, 6.0-8.0 μg/day | 19 (7.9) | 2 (33.3) |  | 18 (7.6) | 3 (42.9) |  |
|  | Past or current tuberculosis | 14 (5.8) | 0 (0.0) | 0.999 | 12 (5.0) | 0 (0.0) | 0.999 |

MET= resting metabolic rate LS-BMAD= Lumbar-spine bone mineral apparent density TBLH-BMC^LBM^=total body less head bone mineral content for lean body mass.

**Supplementary Table 4: Characteristics of participants with complete data for study outcome, tanner stage, orphanhood and for CWH CD4+ T cell count and HIV viral load, compared to those with missing data for one or more variables**

|  | | **Boys**  **N=247** | | | **Girls**  **N=245** | | |
| --- | --- | --- | --- | --- | --- | --- | --- |
|  |  | **Non-missing data**  **n=235** | **Missing data**  **n=12** | **p-value** | **Non-missing data**  **n=231** | **Missing data**  **n=14** | **p-value** |
| **Socio-demographic factors** | Age years, mean (SD) | 13.6 (2.5) | 12.8 (2.4) | 0.266 | 13.8 (2.5) | 12.9 (2.6) | 0.200 |
|  | Socioeconomic status, n(%) |  |  |  |  |  |  |
|  | Tertile 1 (low) | 75 (31.9) | 6 (50.0) | 0.151 | 81 (35.1) | 3 (21.4) | 0.370 |
|  | Tertile 2 (middle) | 78 (33.2) | 5 (41.7) |  | 75 (32.5) | 7 (50.0) |  |
|  | Tertile 3 (high) | 82 (34.9) | 1 (8.3) |  | 75 (32.5) | 4 (28.6) |  |
|  | Orphanhood (one or both parents) deceased^*^ | 60 (25.5) | 2 (16.7) | 0.727 | 62 (26.8) | 3 (21.4) | 0.894 |
| **Pubertal Stage^** | Tanner I | 14 (6.0) | 1 (8.3) | 0.641 | 25 (10.8) | 3 (21.4) | 0.222 |
|  | Tanner II | 91 (38.7) | 7 (58.3) |  | 37 (16.0) | 4 (28.6) |  |
|  | Tanner III | 45 (19.1) | 1 (8.3) |  | 46 (19.9) | 3 (21.4) |  |
|  | Tanner IV | 78 (33.2) | 3 (25.0) |  | 81 (35.1) | 1 (7.1) |  |
|  | Tanner V | 7 (3.0) | 0 (0.0) |  | 42 (18.2) | 3 (21.4) |  |
| **Lifestyle factors** | Physical activity level |  |  |  |  |  |  |
|  | Low, <600 MET mins/week | 82 (34.9) | 3 (25.0) | 0.337 | 125 (54.1) | 8 (57.1) | 0.600 |
|  | Moderate, 600-3000 MET mins/week | 107 (45.5) | 8 (66.7) |  | 88 (38.1) | 4 (28.6) |  |
|  | High, >3000 MET mins/week | 49 (19.6) | 1 (8.3) |  | 18 (7.8) | 2 (14.3) |  |
|  | Daily dietary calcium intake |  |  |  |  |  |  |
|  | Very low, <150 mg/day | 134 (57.0) | 8 (66.7) | 0.490 | 130 (56.3) | 9 (64.3) | 0.223 |
|  | Low, 150-299 mg/day | 47 (20.0) | 3 (25.0) |  | 40 (17.3) | 0 (0.0) |  |
|  | Moderate, 300–450 mg/day | 54 (23.0) | 1 (8.3) |  | 61 (26.4) | 5 (35.7) |  |
|  | Daily dietary vitamin D |  |  |  |  |  |  |
|  | Very low, <4.0 μg/day | 54 (23.0) | 3 (25.0) | 0.096 | 55 (23.8) | 4 (28.6) | 0.161 |
|  | Low, 4.0-5.99 μg/day | 163 (69.4) | 6 (50.0) |  | 158 (68.4) | 7 (50.0) |  |
|  | Moderate, 6.0-8.0 μg/day | 18 (7.7) | 3 (25.0) |  | 18 (7.8) | 3 (21.4) |  |
|  | Past or current tuberculosis | 13 (5.5) | 1 (8.3) | 0.999 | 11 (4.8) | 1 (7.1) | 0.999 |

MET= resting metabolic rate

**Supplementary Table 5: Baseline ART regimens of study participants who were followed-up to one-year**

|  | | **Boys living with HIV**  **N=125** | **Girls living with HIV**  **N= 119** |
| --- | --- | --- | --- |
| **Antiretroviral treatment regimen n(%)** | TDF, 3TC or XTC, LPV/r or ATV/r | 7 (5.6) | 6 (5.0) |
|  | TDF, 3TC or XTC, EFV or NVP | 33 (26.4) | 32 (26.9) |
|  |  |  |  |
|  | ABC, 3TC or XTC, LPV/r or ATV/r | 24 (19.2) | 15 (12.6) |
|  | ABC, 3TC or XTC, EFV or NVP | 3 (2.4) | 9 (7.6) |
|  |  |  |  |
|  | AZT, 3TC or XTC, LPV/r or ATV/r | 7 (5.6) | 9 (7.6) |
|  | AZT, 3TC or XTC, EFV or NVP | 46 (36.8) | 47 (39.5) |
|  |  |  |  |
|  | Missing or incomplete regimen* | 5 | 1 |

ART= Antiretroviral treatment, TDF= tenofovir disoproxil fumarate, 3TC= Lamivudine LPV/r = Lopinavir with boosted ritonavir, ATV/r= Atazanavir with boosted ritonavir, EFV = Efavirenz, NVP= Nevirapine, ABC= Abacavir, AZT= Zidovudine

* Only one or no antiretroviral drugs listed
